# Protistan predation selects for antibiotic resistance in soil bacterial communities

**DOI:** 10.1038/s41396-023-01524-8

**Published:** 2023-10-04

**Authors:** Thi Bao-Anh Nguyen, Michael Bonkowski, Kenneth Dumack, Qing-Lin Chen, Ji-Zheng He, Hang-Wei Hu

**Affiliations:** 1 School of Agriculture, Food and Ecosystem Sciences, Faculty of Science, The University of Melbourne, Parkville, VIC 3010 Australia; 2 Terrestrial Ecology, Institute of Zoology, University of Cologne, Köln, Germany

**Keywords:** Soil microbiology, Antibiotics, Applied microbiology

## Abstract

Understanding how antibiotic resistance emerges and evolves in natural habitats is critical for predicting and mitigating antibiotic resistance in the context of global change. Bacteria have evolved antibiotic production as a strategy to fight competitors, predators and other stressors, but how predation pressure of their most important consumers (i.e., protists) affects soil antibiotic resistance genes (ARGs) profiles is still poorly understood. To address this gap, we investigated responses of soil resistome to varying levels of protistan predation by inoculating low, medium and high concentrations of indigenous soil protist suspensions in soil microcosms. We found that an increase in protistan predation pressure was strongly associated with higher abundance and diversity of soil ARGs. High protist concentrations significantly enhanced the abundances of ARGs encoding multidrug (*oprJ* and *ttgB* genes) and tetracycline (*tetV)* efflux pump by 608%, 724% and 3052%, respectively. Additionally, we observed an increase in the abundance of numerous bacterial genera under high protistan pressure. Our findings provide empirical evidence that protistan predation significantly promotes antibiotic resistance in soil bacterial communities and advances our understanding of the biological driving forces behind the evolution and development of environmental antibiotic resistance.

## Introduction

Antibiotic resistance represents one of the most significant global health challenges of the 21st century, with the worldwide spread of antibiotic resistance potentially causing up to 10 million deaths by 2050 if timely action is not taken [1]. However, bacterial antibiotic resistance is a natural phenomenon that has emerged and evolved over millions of years, predating the antibiotic era of humans [2]. Resistance determinants, including antibiotic resistance genes (ARGs or “resistome”) and antibiotic-resistant bacteria (ARB), have been found in remote isolated human bodies or caves, as well as ancient permafrost across all continents on our planet [2, 3]. Soils, which are the richest habitats of microorganisms, are a significant source of antibiotics and antibiotic-producing bacteria [4, 5]. Most of the antibiotics used in human and animal disease control originate from natural isolates found among soil bacteria or fungi [5]. Despite this knowledge, we still lack a mechanistic understanding of the key driving forces causing the emergence and evolution of ancient and ongoing antibiotic resistance in natural soil settings, which hampers our ability to predict and mitigate antibiotic resistance under future scenarios of global change.

In natural habitats, bacteria face a wide range of biotic and abiotic stressors including competition, predation and changes in abiotic environmental conditions (e.g., pH, temperature or salinity) [6, 7]. Since bacteria lack the capacity and energy to deal with those stressors independently, bacteria have developed key strategies, such as the production of antibiotics and/or the evolution of the antibiotic resistance system to effectively withstand these challenges. The development of ARGs, which typically encode antibiotic deactivation, cellular protection or efflux pump machineries, empowers bacteria to resist the toxicity of antibiotics and other bacteriocins released by themselves or competitors. Bacteria frequently encounter protists, their primary predators in the soil matrix. The majority of soil protists are bacterivores, and bacteria evolved sophisticated defense strategies [8]. Soil protists exert a significant selection pressure on soil bacterial communities, because they avoid antibiotics-producers and selectively consume their non-defended bacterial prey [9]. These predator - prey interactions have been postulated as a potential driver of antibiotic resistance in ruminants [10]. However, whether indigenous soil protists also affect the antibiotic resistance of bacterial communities in soil remains elusive. Therefore, we hypothesize that soil protists, as the key predators of soil bacteria, play an important role in driving the abundance and diversity of antibiotic resistance in soil bacterial communities.

**Fig. 1:**
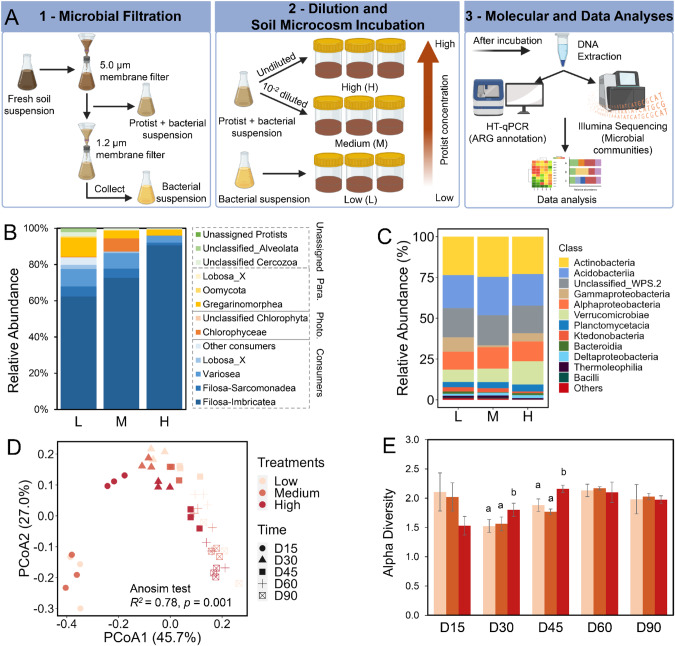
Experiment workflow of this study and the overall profile of soil protist and bacterial communities. **A** Experiment workflow of this study. Firstly, we obtained indigenous (i) protist and bacterial suspension and (ii) bacterial suspension from a forest soil through a series of filtration. Secondly, we created a gradient of three protist concentrations (low, medium and high) by diluting the collected protist and bacterial suspensions, and then inoculated them into sterilized forest soil samples. The soil microcosms were incubated at 25 °C for 90 days. Thirdly, soil samples were destructively collected at days 0, 15, 30, 45, 60, and 90, and then DNA was extracted for high-throughput quantitative PCR (HT-qPCR), qPCR and Illumina amplicon sequencing to characterize the profiles of ARGs and microbial communities. **B** Relative abundance of soil protists at the class level and trophic functional group in protist treatments across all time points. **C** Relative abundance of bacteria at the class level in protist treatments across all time points. **D** Principal coordinate analysis showing differences in bacterial community composition at different protist treatments over time. **E** Temporary alteration in alpha diversity (Shannon index) of bacteria over the incubation time. Letters indicate the significant difference in alpha diversity among different treatments in each time point (one-way ANOVA, post hoc LSD test, *p* < 0.05). Photo. Phototrophs, Para. Parasites, L Low, M Medium, H High protist concentrations.

To test our hypothesis, we assessed the influence of soil protists on the ARGs and bacterial community by establishing soil microcosm incubations using isolated indigenous protists (a size range of 1.2–5.0 µm) and bacterial communities. We collected the indigenous bacterial and protist communities from a forest soil that had limited anthropogenic disturbance using a filtration approach. We then established the soil microcosms by inoculating a gradient of three protist concentrations (low, medium and high) into sterilized indigenous soils for a period of 90 days (Fig. 1A). We used high-throughput quantitative PCR analysis and amplicon sequencing to characterize the profile of ARGs and bacterial community in response to varying protist concentrations.

## Materials and methods

### Soil sampling

We collected forest soil samples from Bunyip State Park (37°58'59.7“S 145°38'48.4“E), Victoria, Australia, at a depth of 0–10 cm, which served as a presentative sample of natural soils with minimal human impacts. Soil samples were transported on dry ice to the laboratory and were divided into three portions for microbial filtration, soil physicochemical analysis and soil microcosm incubation. All soil samples were sieved through a 2 mm mesh to remove roots, plant residues, macrofauna and stones before analysis of soil basic properties [11]. The forest soil was a loamy sand with a soil pH of 5.21 and a soil moisture content of 8.25%. The total carbon and nitrogen contents were 3.48% and 0.16%, respectively.

### Soil microcosm incubation study

To obtain indigenous soil protist (size ranging 1.2–5.0 µm) and bacterial communities, we added 150 g of fresh soil (equivalent dry weight) and 300 mL of sterile distilled water into a 1 L Erlenmeyer flask. We mixed the contents well for two hours at 25 °C in the dark to create soil microbial suspensions. We then centrifuged soil microbial suspensions at 1500 × *g* for 10 min, and collected the supernatants to obtain a mixture of protist and bacterial suspension and only bacterial suspension, using a series of filtration steps [12–14] (Fig. 1A). Firstly, a 5.0 µm membrane filter was used to remove soil fauna and soil particles, and more importantly, to collect the suspension of both protist and bacteria. Secondly, a half of the protist and bacterial suspension was further filtered through a 1.2 µm membrane filter to remove all protists and fungi, and to collect the bacterial suspension. We confirmed the absence of protist contamination in the collected bacterial suspension by examining it under an inverted phase-contrast microscope (Olympus CKX53, Tokyo, Japan).

The soil microcosm incubation was established with three protist treatments (three replicates per treatment) by inoculating with a gradient of three protist concentrations, including low (L; inoculated with only the bacterial suspension); medium (M; inoculated with 10^-2^-diluted protist and bacterial suspension), and high treatment (H; inoculated with undiluted protist and bacterial suspension) (Fig. 1A). The experiment was conducted using 1 L glass jars containing 250 g soils (equivalent dry weight) sterilized by γ-radiation (50 kGy), and pre-incubated for 16 h with 3 mg of the fungal inhibitor cycloheximide (CAS Number: 239763; Merck, Australia) per gram of dry soil to eliminate any potential fungal effects [15]. Each soil microbiome in the microcosms was inoculated with 10 mL of the indigenous protistan and bacterial suspensions. Soil moisture content was maintained at the original moisture condition (8.25%) of the forest soil. The microcosms were incubated for 90 days at 25 °C in the dark, and the soil microcosms were loosely covered to maintain aerobic conditions. Sterile water was supplemented every three days to maintain soil moisture content. Soil samples were collected at six time points, namely days 0 (for supplementary data), 15, 30, 45, 60 and 90, for molecular analysis including high-throughput quantitative PCR (HT-qPCR), qPCR and Illumina sequencing to characterize bacterial and protist communities.

### DNA extraction and high-throughput quantitative PCR analysis of ARGs

Soil genomic DNA was extracted from 0.25 g of soil samples using the Powersoil DNA Isolation Kit (MoBio Laboratories, Carlsbad, CA, USA) following the manufacturer’s instructions. The extracted DNA was evaluated for quantity and purity using the NanoDrop spectrophotometer (ND2000c, NanoDrop Technologies, Wilmington, DE, USA). To quantify ARGs encoding resistance to all main antibiotic classes, the HT-qPCR was performed using 285 primer sets on a Wafergen Smart-Chip Real-TimeSystem (Fremont, CA, USA) (Table S1) [16]. SensiMix SYBR No-ROX reagent (CAS Number: QT650-05; Bioline, Australia), primers, and extracted DNA were added to make the 100 nL reaction mixture. Positive and negative controls were 16S rRNA gene and sterilized water, respectively. HT-qPCR amplifications were performed in technical triplicates per sample under the following conditions: denaturation for 10 min at 95 °C, followed by 40 cycles of 95 °C for 30 s and 60 °C for 30 s. The results were evaluated using the comparative C_T_ method [16] based on three main criteria: (1) the detection limit was a threshold cycle (C_T_) of 31; (2) positive results of triplicates should not be below the detection limit; and (3) amplicons with multiple melting curves were ruled out. We calculated relative copy number using the following equation: relative gene copy number = $10^{(31-{{{\rm{C}}}}_{{{\rm{T}}}})/(10/3)}$, where C_T_ refers to the HT-qPCR results [17]. The relative abundance of ARGs was determined by taking the log-transformation of the relative copy numbers of ARGs as the output of the HT-qPCR.

### Characterization of soil microbial communities

The absolute abundance (i.e., copy gene number per g soil) of 18S rRNA genes (protists) and 16S rRNA genes (bacteria) across different time points (days 0, 15, 30, 45, 60 and 90) was quantified by qPCR using the qPCR primer sets including TAReuk454FWD1/TAReukREV3 [18] and universal eubacterial primers 1132 F/1108 R, respectively [19, 20]. The qPCR of 16S rRNA genes was performed in triplicate 20 μL reactions containing SYBR green master mix (Applied Biosystems, USA), 2 μL DNA template and primers, using a thermocycler program of 40 cycles of 15 s at 95 °C and 60 s at 60 °C [19]. For protists, the qPCR of 18S rRNA genes was amplified in triplicate 20 uL reactions under the following thermocycle condition: an initial denaturation at 95 °C for 5 min; followed by 10 cycles of 94 °C for 30 s, 57 °C for 45 s (annealing) and 72 °C for 60 s (extension); and followed by 25 cycles of 94 °C for 30 s, 48 °C for 45 s (annealing), and 72 °C for 60 s (extension); the last step being extension at 72 °C for 2 minutes [18]. To generate standard curves for qPCR analysis, PCR amplicons of the protist 18S rRNA gene and bacterial 16S rRNA gene using the primers TAReuk454FWD1/TAReukREV3 and 1132 F/1108 R, respectively, were purified and ligated into the pGEM-T Easy vector (Promega, Madison, WI, USA), and the resultant ligation products were transformed into JM109 competent cells following the manufacturer’s instructions. The resulting clones containing the targeted 18S rRNA gene and 16S rRNA gene fragments were selected to extract plasmid DNAs, and standard curves were generated by preparing 10-fold serial dilutions of the plasmids.

Bacterial and protist communities in raw soil (three replicates) before the incubation and soil samples collected during the incubation were characterized using the primer sets 515 F/806 R [21] and TAReuk454FWD1/TAReukREV3 [18] to amplify 16S rRNA gene and eukaryotic 18S rRNA gene, respectively (Table S1). The amplicons were cleaned, measured and pooled into an equimolar pool before being quantified using a High-Sensitivity D1000 Tape on an Agilent 2200 TapeStation. Amplicon sequencing was performed following the manufacturer’s protocol of 2 × 300 bp paired end on a MiSeq sequencer (Illumina; San Diego, CA, USA) at Australian Genome Research Facility, Australia. The demultiplexed raw reads were primer trimmed and quality-filtered using the “cutadapt” plugin and the “DADA2” software package [22]. Microbial profiling was performed using QIIME 2 [23]. Taxonomy classification of the amplicon sequence variant (ASV) sequences was conducted using the “q2-feature-classifier” plugin of QIIME 2 [24]. The latest Protist Ribosomal Reference (PR2) database (v 5.0.1) [25] and SILVA database (v138) [26] were used for assigning taxonomy to protists and bacteria, respectively. Protists (excluding plants, metazoan and fungi) were classified into three trophic functional groups: consumers, phototrophs and parasites [27], with undetermined functional lineages named as “unassigned protists”.

### Statistical analyses

Most of graphs in this study were visualized using the “ggplot2” [28] and “VennDiagram” [29] packages in R, with the exception of the co-occurrence network visualization which was created using Cytoscape (https://cytoscape.org/). The number of unique and shared ARGs was calculated and visualized using the package “VennDiagram” [29]. The bacterial community composition in different treatments over time was analyzed using principal coordinate analysis based on the Bray-Curtis distance and Anosim test in the package “vegan” [30]. The compositions of bacterial and protist communities and ARGs were represented by the first axis of the principal coordinate analysis (PCoA1) for subsequent analyses. The significant differences in the relative abundance and diversity of ARGs and bacterial community among different treatments were estimated by one-way analysis of variance (ANOVA, post hoc LSD test, *p* < 0.05) in SPSS (version 28.0.1.1). Two-way ANOVA was performed to evaluate the effects of different factors (protist concentrations, incubation time and their interactive effects) on the abundance and number of ARGs, as well as the bacterial community composition and diversity.

To assess the importance of protist concentrations and community composition on bacterial communities and sampling time in shaping the bacterial composition and number of ARGs, random forest modellings with 5,000 trees per model was performed using the “randomForest” package [31]. The significance of tested factors (i.e., the percentage increase of mean squared error - %IncMSE; *p* < 0.01) was confirmed using the “A3” package in R. The co-occurrence network analysis was constructed based on Spearman’s correlation with correlation coefficients of ≥ 0.3 and ≤ −0.3 (all *p* values < 0.001) among affected ARGs, protist and bacterial genera across all samples. This analysis included three replicates per treatment, resulting in a total of 15 replicates at five time points, and then visualized in Cytoscape [32]. To unravel bacteria-bacteria interaction upon different protist concentrations, we identified the strong associations among bacterial genera in each treatment through Spearman’s correlation (robust coefficients of ρ ≥ 0.65 and ρ ≤ −0.65, *p* < 0.001).

## Results

### Protist community and their effect on bacterial community

The majority of protists were consumers, accounting for 89% of total protist sequences (Fig. 1B), while phototrophs and parasites constituted only a minor proportion. Notably, the omnivorous Cercozoa (77% of total protist abundance on average) were found to be the most abundant consumers (Fig. S1A). Protistan consumers dominated all treatments over time (Fig. S1B). A similar pattern was observed in the community composition of protists in raw soil (Fig. S2A, B), where consumers accounted for 77% of the total protist relative abundance, with the phyla Cercozoa (40%) and Lobosa (23%) being the most prevalent (Fig. S2A-B). We also observed the higher copy number of 18S rRNA genes in the beginning of the incubation period (day 0 and 15) in high protist concentration than low and medium protist treatments (Fig. S3A), whereas the alpha diversity of protists was insignificantly changed among different treatments over time (Fig. S3B). The highest abundance of protists was observed on day 15. Bacterial community was dominated by the class *Actinobacteria* across all treatments like the raw soil (Fig. S3C), followed by *Acidobacteriia*, unclassified *WPS.2* and *Gammaproteobacteria* (Fig. 1C). On day 0, the relative abundance of bacteria was insignificantly differed among protist treatments, but the 16S rRNA gene number was increased from day 15 to 90 with a higher abundance in higher protist treatment at days 15 and 30 (Fig. S3D).

We characterized changes in the diversity and compositions of the bacterial community across the three protist treatments. The bacterial community composition and diversity were significantly affected by protist concentrations, particularly on days 30 and 45 (Figs. 1D, E and S4). Principal coordinate analysis revealed that a substantial 78% of the variation in the bacterial community composition (Anosim test, *R*^*2*^ = 0.78; *p* = 0.001; Fig. 1D) was explained by the strong influence of soil protists over time. The diversity of the bacterial community was higher when exposed to the higher predatory pressure of soil protists in the middle of the incubation period (days 30 and 45), compared to low and medium protist levels (Fig. 3B). Protists also enriched the relative abundances of bacteria in the high concentration treatment at days 15, 30 and 45 (Figs. S3D and S4). We observed an increase in the abundance of many bacterial genera belonging to dominant classes *Acidobacteriia*, *Actinobacteria*, *Alphaproteobacteria* and *Planctomycetacia* across different time points, especially among antibiotic-producing bacteria (APB) such as *Mycobacterium*, *Nocardia* and *Streptomycetaceae* (Fig. S4). Furthermore, we found that the high protist levels increased negative associations among bacterial genera, with 5.1% and 7.9% at low and medium compared to 39.1% at high protist levels, respectively (Fig. S5).

### Increasing protist concentrations increased the ARG richness and relative abundance

**Fig. 2:**
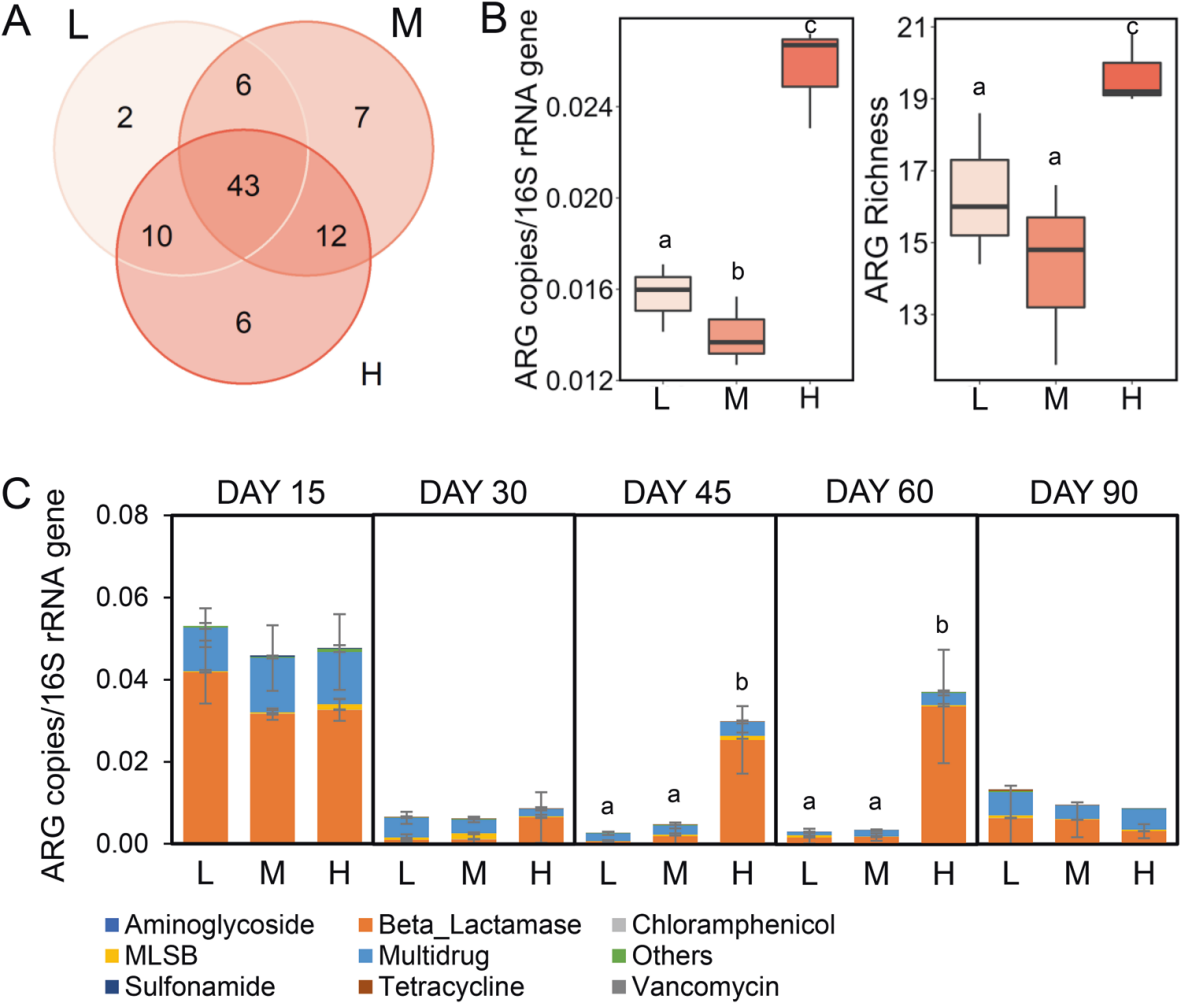
High protist concentration increased ARG richness and relative abundance. **A** Unique and shared ARGs among the different protist treatments. **B** Mean relative abundance and richness of ARGs in different soil samples across all time points. **C** Changes in the relative abundance of ARGs at different incubation days. Letters indicate the significant difference in the ARG profile among treatments (one-way ANOVA, post hoc LSD test, *p* < 0.05). L Low, M Medium, H High protist concentrations).

Across all samples, we detected a total of 82 unique ARGs encoding resistance to major known antibiotics (Fig. S6), with many unique ARGs detected under the higher grazing pressure of protists (Fig. 2A). Beta-lactamase and multidrug resistance genes were the most prevalent types of ARGs across all samples (Figs. 2C–S7A). We found that the relative abundance and richness (i.e., number) of ARGs were significantly higher in the high protist treatment (0.03 and 19.67 on average, respectively), compared to the lower protist treatment (Fig. 1B; LSD test, *p* < 0.05). The ARG richness was enhanced over the incubation time, compared to day 0 (Fig. S7B). In particular, increasing protist concentrations considerably increased the abundance of ARGs conferring resistance to beta-lactamase, multidrug and (macrolide, lincosamide, and streptogramin B resistance) MLSB at days 45 and 60 (Figs. 1C; S6). A similar pattern was observed in the number of ARGs at day 60 (Fig. S7B). We found no significant differences in the ARG profiles between the low and medium protist treatments (Figs. 1C; S7).

**Fig. 3:**
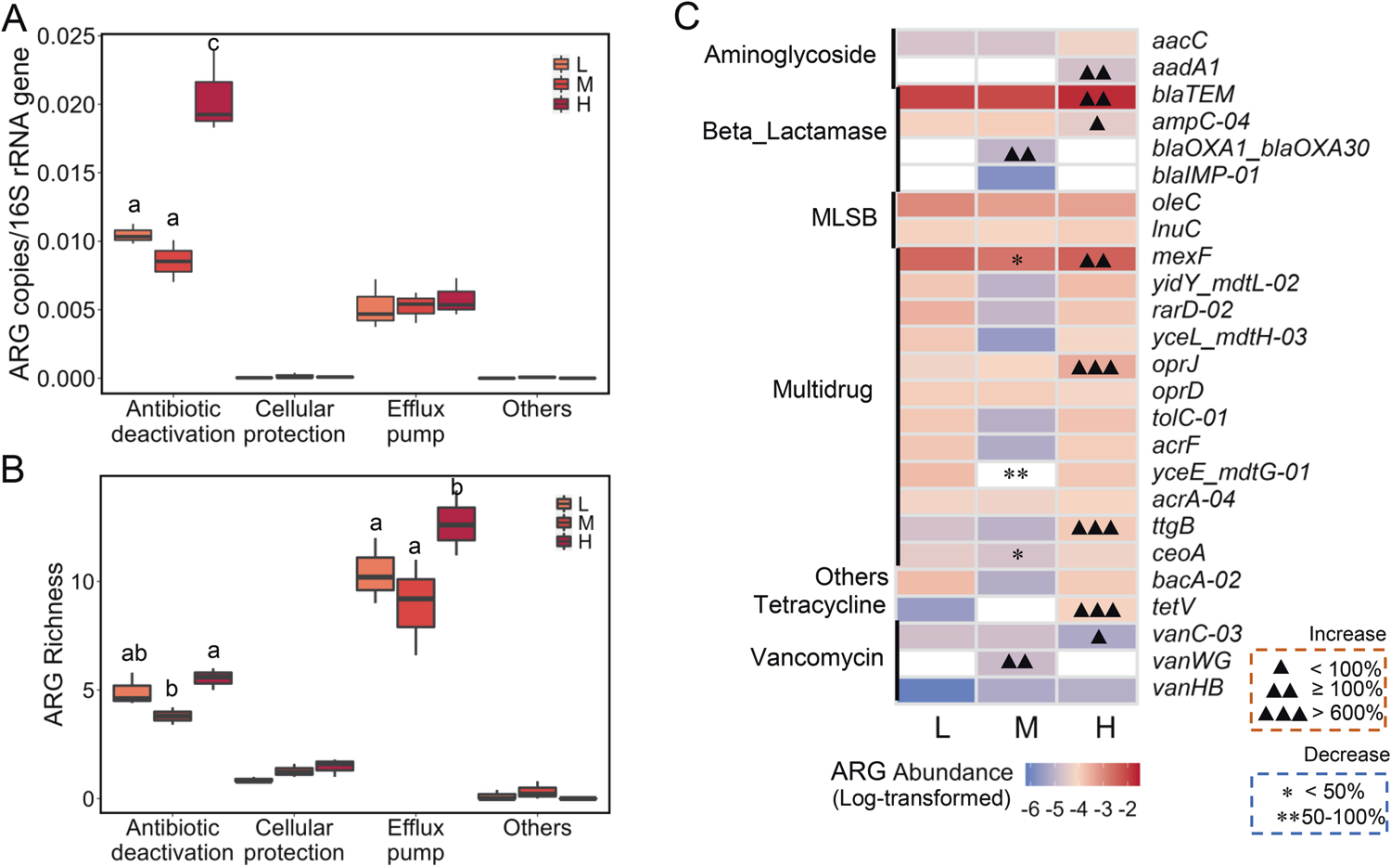
Effects of protists on the relative abundance and richness of ARGs involved in antibiotic resistant mechanisms. **A** The relative abundance and (**B**) richness of ARGs classified according to their different antibiotic resistant mechanisms. Letters indicate the significant difference in the ARG profile among treatments (one-way ANOVA, post hoc LSD test, *p* < 0.05). **C** The abundance of dominant ARGs and their changes across the three protist treatments. Triangles and asterisks indicate the abundance increase and decrease of ARGs in the medium and high protist treatments (unit: percentage (%)), respectively, compared to the low protist treatments. L Low, M Medium, H High protist concentrations.

The high protist concentration treatment significantly increased the abundance of ARGs encoding antibiotic deactivation and the ARG number encoding efflux pump (Figs. 3A, B; one-way ANOVA, LSD test, *p* < 0.05). In high protist treatments, the abundances of genes encoding multidrug (*oprJ* and *ttgB* genes) and tetracycline (*tetV)* efflux pump were enriched by 608%, 724% and 3052%, respectively, compared to the low treatments (Fig. 3C). Aminoglycoside (*aadA1*) and beta-lactamase resistance genes (*blaTEM* and *ampC-04*) conferring antibiotic deactivation were enriched in the medium and high protist treatments, but several multidrug resistance genes (*mexF*, *yceE_mdtG-01* and *ceoA*) decreased their abundances in the medium protist treatment. A similar pattern was detected for the *vanWG* and *vanC-03* genes functioning cellular protection by altering antibiotic target sites [33, 34].

### Protists were strongly associated with soil resistome

**Fig. 4:**
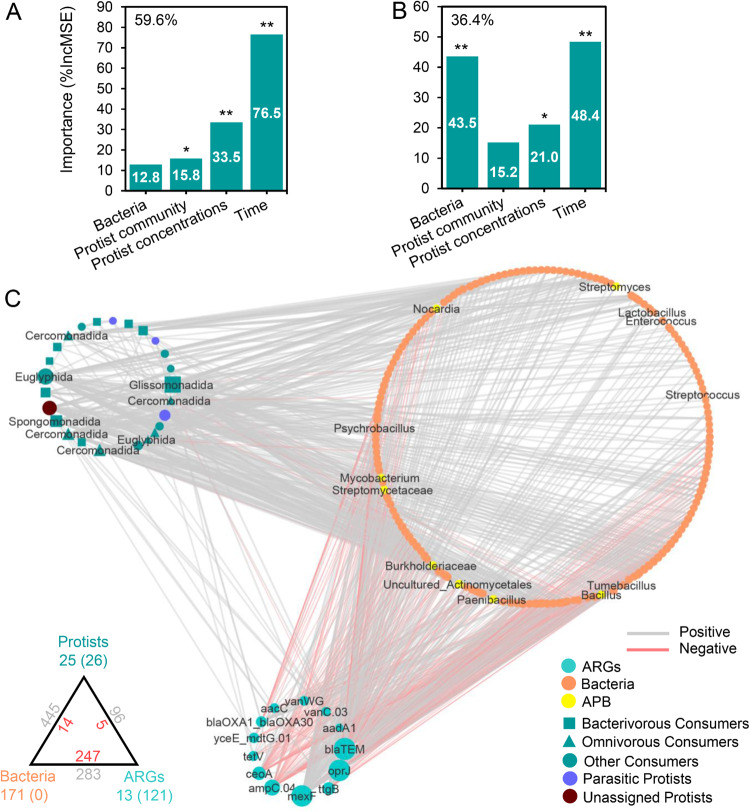
Protists were strongly associated with soil resistome and bacteria. The importance (i.e., percentage increase of mean squared error - %IncMSE) of different factors on the ARG composition (**A**) and richness (**B**), analyzed by random forest modelling. Asterisks indicate the significant importance of factors: *p < 0.05; **p < 0.01. **C** Co-occurrence network showing the association of affected ARGs with bacterial and protist genera. Each node represents a taxon at the genus level. The node of protists is displayed at the order level. Size of node is proportional to the number of connections. Thickness of edges is proportional to Spearman correlation coefficients, divided into positive (ρ ≥ 0.3, *p* < 0.001; grey) or negative (ρ ≤ −0.3, *p* < 0.001; red) edges. A summary of node–edge statistics is provided at the bottom left of the network. Colored numbers represent the number of nodes belonging to the corresponding category. Numbers in backets denote the number of inner connections. The grey and red numbers adjacent to edge connections represent positive and negative cross-group interaction. APB: potential antibiotic-producing bacteria.

We further estimated the effects of protists on bacterial community and ARGs. Random forest analysis revealed that the incubation time, protist concentrations and community were important predictors of the ARG composition, explaining 59.6% of the variation (Fig. 4A). Moreover, the ARG richness was influenced by bacterial community (i.e., beta diversity), protist concentrations and incubation time (Fig. 4B). We then explored the relationships among protists, ARGs and bacteria using the co-occurrence network analysis (Fig. 4C). We found that 171 bacterial and 21 protistan consumer genera were significantly associated with affected ARGs. The network analysis revealed positive connections between many protistan genera of the orders Glissomonadida and Spongomonadidae (bacterivores), and Euglyphida and Cercomonadida (omnivores), with bacterial genera, particularly APB such as *Bacillus*, *Streptomyces*, *Mycobacterium*, *Nocardia* and *Streptomycetaceae*. Furthermore, we found robust correlations between enriched ARGs and specific protistan genera, including *aadA1* with bacterivorous *Allantion* and *Allapsidae* (Glissomonadida); *ttgB* with bacterivorous *Spongomonas* (Spongomonadidae)*; oprJ* with undetermined Cercozoa; *tetV* with *Allantion* (Glissomonadida); and *vanWG* with *Sandonidae* (Glissomonadida) and *Acanthamoeba* (Centramoebida). The most abundant gene, *blaTEM*, which encodes antibiotic deactivation, was negatively associated with bacterivorous *Cercomonadidae* (Cercomonadida).

## Discussion

Our study investigated the role of soil protists in the development of the soil resistome, which is important for understanding the spread of antibiotic resistance in natural environments. We found that the enrichment of natural undiluted protistan inoculants exerted a high pressure on bacterial communities. Protists significantly enriched the profile of ARGs, including those conferring resistance to widely used antibiotics such as beta-lactams, aminoglycosides, tetracyclines and multidrug resistance. Our results also showed that the overall bacterial community composition and the relative abundance of specific bacterial taxa were changed by the grazing pressure of protists. Overall, our study provides compelling evidence that soil protists play a crucial role in driving the development of the bacterial antibiotic resistance in natural habitats, and highlights the need to consider these predators in efforts to mitigate the spread of antibiotic resistance.

Our results support the hypothesis that soil protists enhance the development of soil ARGs at the community level by significantly influencing both the composition and functions of bacterial communities. Firstly, the higher grazing pressures of protists, especially the predominance of omnivorous or bacterivorous Cercozoa (Fig. S1A) may trigger antimicrobial excretion by bacteria to selectively suppress protists [35]. We observed a strong enrichment of ARGs encoding the deactivation of antibiotics (aminoglycoside and beta-lactamase) and multiple efflux pump in our study (Fig. 3C), indicating that bacteria are producing antibiotics as selection pressure proliferate ARB and ARGs under the grazing pressure of protists [36], thereby enhancing the soil resistome. Previous in vitro experiments have reported that specific antibiotics (e.g., violacein, pyrrolnitrin, massetolide, and viscosin) produced by bacterial strains can kill some specific protist lineages [37, 38]. Additionally, certain bacterial taxa as antibiotic producers, e.g., *Streptomyces*, *Bacillus*, become resistant to grazing while other grazing-susceptible and antibiotic-susceptible bacterial taxa are preferably consumed by predatory protists. These predator-prey interactions could explain negative associations between susceptible bacterial genera and protistan consumers (Fig. 4C). We recorded the increasing abundance of potential APB *Mycobacteria*, *Nocardia* and *Streptomycetaceae* which were positively correlated with consumers. Secondly, harsh conditions (i.e., high protist grazing pressure, carbon-source reduction and limited space) could increase bacterial competition, inducing antibiotic production to kill others [39]. Our results indicated an increase in the negative bacterial interaction with increasing protist concentrations (Fig. S5), suggesting enhanced protist-induced competition among bacteria. Thirdly, protists might promote bacterial activity and growth [40], as supported by the increasing abundance of bacteria taxa in high protist treatments and the positive protist-bacterial relationship.

We found the highest abundance of ARGs in all treatments on day 15 (Fig. 2C) during the exponential growth of bacteria recolonizing the sterilized soil (Fig. S3D). Subsequently, the ARG abundance declined, except in treatments with high protist levels at the peak bacterial abundance on both days 45 and 60 (Fig. 2C). The peak abundance of ARGs on day 15 coincided with the highest abundance of protists and, therefore, likely reflects the strongest predation pressure (Fig. S3A). At this point, competition between bacterial taxa would also be maximized due to the peak density and decreasing nutrient resources (days 45 and 60). Both the high protist abundance and intense bacterial competition at the peak density act as strong selection pressures, inducing bacteria to produce antimicrobials to combat protists or compete with other bacteria. The antimicrobials produced by bacteria can inhibit or kill protists or bacterial competitors by influencing their cellular processes, such as cell lysis, encystation, cyst reactivation, paralysis, or growth restriction of protist cells [41, 42], and causing cell lysis or growth inhibition of susceptible bacteria [43, 44]. Jousset et al. (2006 and 2010) demonstrated that bacterial strains *Pseudomonas fluorescens* CHA0 and Q2-87 produced antibiotics 2,4-diacetylphloroglucinol (DAPG), pyoluteorin and pyrrolnitrin to impede protist growth or repel the protists’ predation under in vitro conditions [37, 38]. The antimicrobials produced by bacteria in all three treatments acted as selection pressures, leading to the enrichment of antibiotic resistant bacteria and proliferation of ARGs within the bacterial community [37, 38, 45]. This finding provides an explanation for the observed highest abundance of ARGs on day 15. As the incubation period progressed, a reduction in nutrient sources and a decreasing relative abundance of protists might have contributed to the decline in the ARG abundance from day 30 to day 90 (Fig. 2C). However, in treatments with high protist levels, the higher abundance of protists, especially omnivorous protists, might exert a pressure on specific bacterial taxa (Fig. S1), which explains the higher ARG abundance at days 45 and 60.

Our study revealed the significant importance of soil protists in shaping the profile of soil ARGs, particularly those involved in multidrug efflux pump and antibiotic deactivation – two of the most common and ancient mechanisms of antibiotic resistance found in soils worldwide [46]. We observed the significant enrichment of ARGs encoding multidrug efflux pumps (*oprJ* and *ttgB* genes) and tetracycline efflux pump (*tetV)* in soil treatments with high protist levels. These efflux pump genes can transport specific or multiple antibiotics out of bacterial cells [47, 48], and are evolutionally ancient resistome (e.g., *oprJ*) found in pristine environments without anthropogenic disturbance [49, 50]. In addition, we detected a significant increase in the abundance of ARGs (aminoglycoside and beta-lactamase) conferring the antibiotic deactivation in the medium and high protist treatments. Antibiotic deactivation is a primary resistance mechanism of action, which inactivates antibiotics by modification through adding a chemical group to antibiotics such as aminoglycosides, or degrading drugs using enzymes such as β-lactamases [47, 51]. A similar pattern was also detected in *vanWG* and *vanC-03* genes which provide cellular protection by altering antibiotic target sites [33, 34]. Most of these ARGs are globally ancient and dominant in various ecosystems [46], highlighting protists as crucial drivers in the development of soil ARGs. In particular, the high protist level with the reduction of carbon sources in soil over time might suppress a selection pressure on dominant bacterial taxa and antibiotic producers, concomitantly intensifying bacterial competition within the community. This also facilitates subdominant or rare bacterial taxa and ARB to grow, thereby explaining the high abundance and number of ARGs under high pressure of protists at days 45 and 60.

The top-down control, including the predation and the presence, of protists can be as a selection pressure for the development of bacterial antibiotic resistance. Predation by protists is a primary regulator of bacterial community structure and lead to the decreasing abundance and richness of specific bacterial taxa [12, 52]. To avoid the predation, bacteria have evolved various anti-predatory strategies, including biofilm formation, changes in size, shape and motility, and the production of antibiotics and other toxins [41, 53, 54]. Among these strategies, the production of toxic antimicrobials is considered as a crucial defensive mechanism [41, 44]. For example, certain bacterial species, such as *Pseudomonas fluorescens* CHA0 [38], have been shown to produce antibiotics to inhibit or kill protists, in response to grazing pressure. The predation pressure exerted by the amoeba *Acanthamoeba castellanii* was found to impose a stronger selection pressure than the competition among *P. fluorescens* CHA0, leading to the production of the antibiotic DAPG. *P. fluorescens* wild strains are more resistant to predators and competitors than non-DAPG-producing mutant bacteria [44]. These toxic compounds can also function as weapons against the protistan predation and the competition posed by bacterial or fungal competitors in soils, thereby acting as a selection pressure that promotes the spread of ARGs and ARB [41]. Both APB and non-APB can develop resistance genes, such as permeability barrier or efflux pump genes, to protect themselves from their own antibiotics or those produced by other strains [55]. These intrinsic resistance genes of bacterial strains are particularly activated and proliferated to defend against the protistan predation, as they shield the cells from the toxic compounds by swiftly pumping them out of the cells [47]. Our work has revealed a strong increase in ARGs encoding multidrug and tetracycline efflux pumps under the increasing pressure of soil protists. Another potential reason for the increase in ARGs in soils is lateral gene transfer among undigested bacteria inside food vacuoles of protists under SOS response [56].

In summary, our study provides novel evidence that soil protists, which are often overlooked, play a critical role in inducing the development of antibiotic resistance in bacterial communities at the community level. This work advances our understanding of the complex biological interactions that underlie the evolution of antibiotic resistance in natural environments and highlights the need for further research to explore the mechanisms by which protists exert selection pressure on bacterial population. By shedding light on the role of soil food web interactions in the spread of antibiotic resistance, our study has important implications for efforts to mitigate the growing threat of antibiotic-resistant infections.

### Supplementary information

Supplementary file

Figure S1

Figure S2

Figure S3

Figure S4

Figure S5

Figure S6

Figure S7

Table S1

## Data Availability

All raw sequencing data was deposited to NCBI Sequence Read Archive (SUB13857267) under the BioProject number PRJNA1019872.
