# Supplementary material for: Protistan predation selects for antibiotic resistance in soil bacterial communities
Source: ISME J. 2023 Oct 4;17(12):2182–9. doi: 10.1038/s41396-023-01524-8 (PMC10689782; doi:10.1038/s41396-023-01524-8)

***Supplementary file***

**Protistan predation selects for antibiotic resistance in soil bacterial communities**

Thi Bao-Anh Nguyen^1,^*, Michael Bonkowski^2^, Kenneth Dumack^2^, Qing-Lin Chen^1^, Ji-Zheng He^1^, Hang-Wei Hu^1,^*

^1^ School of Agriculture, Food and Ecosystem Sciences, Faculty of Science, The University of Melbourne, Parkville VIC 3010, Australia.

^2^ Terrestrial Ecology, Institute of Zoology, University of Cologne, Köln, Germany.

* For correspondence:

Thi Bao Anh Nguyen; Email: [thibaoanhn@unimelb.edu.au](mailto:thibaoanhn@unimelb.edu.au).

Hang-Wei Hu, Email: [hang-wei.hu@unimelb.edu.au](mailto:hang-wei.hu@unimelb.edu.au).

Address: School of Agriculture, Food and Ecosystem Sciences, Faculty of Science, The University of Melbourne, Parkville VIC 3010, Australia.

**Figure S1.** (A) Mean relative abundance of protistan consumers at the order level belonging to the dominant phylum Cercozoa across all time points. Asterisks indicate significant differences in the abundance of protist or bacterial taxa compared to control treatments (one-way ANOVA, post hoc LSD test, *p* < 0.05). (B) Relative abundance and community composition of protists across different time points. (Abbreviations: Photo. = phototrophs; Para. = parasites; L = low; M = medium; H = high protist concentrations).


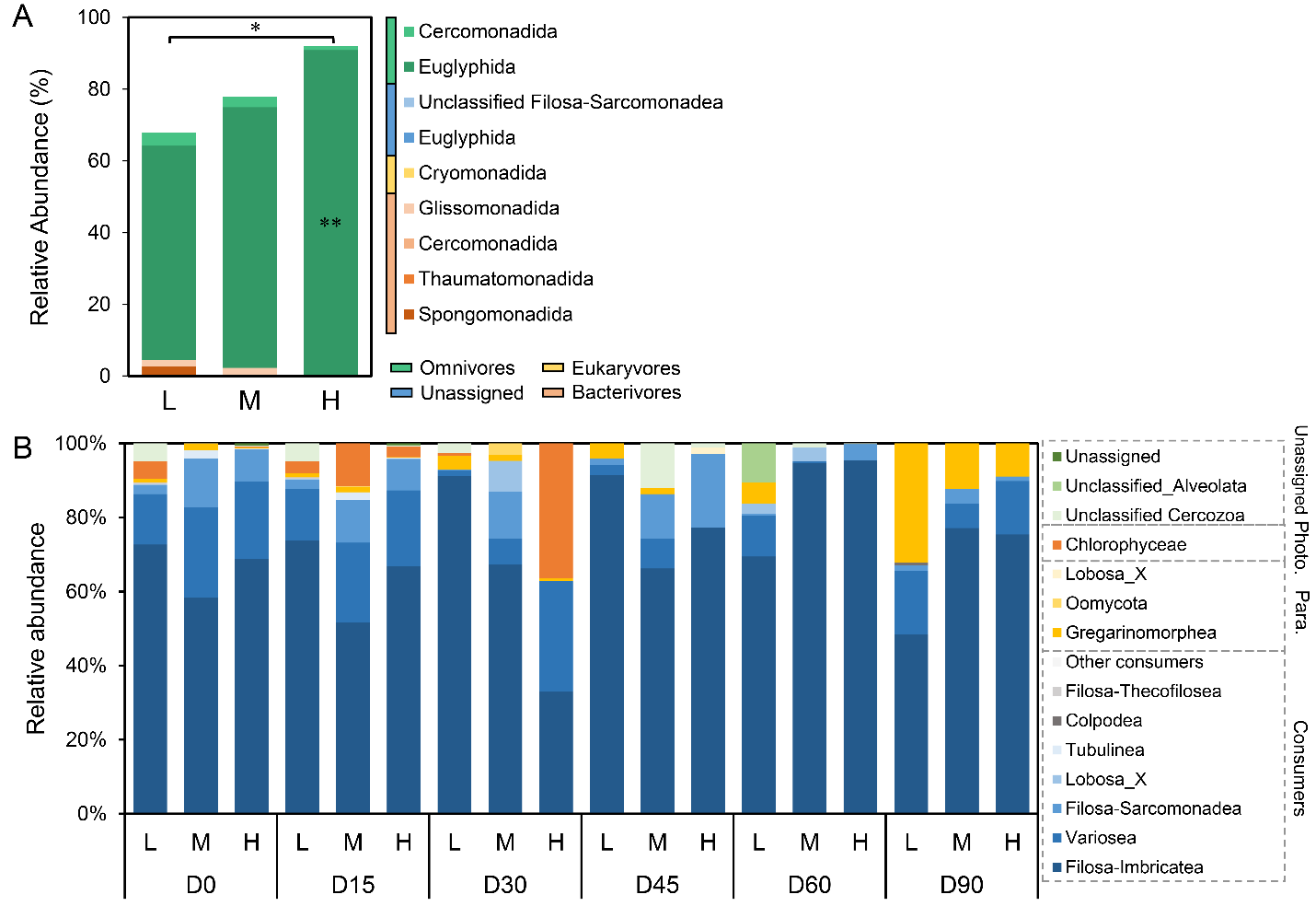


**Figure S2. Relative abundance of protist and bacterial communities in raw soil.** (A) Relative abundance and composition of soil protists at the class level and trophic functional group. **(B)** Relative abundance of soil protists at the phylum level. (Abbreviations: Photo. = phototrophs; Para. = parasites).


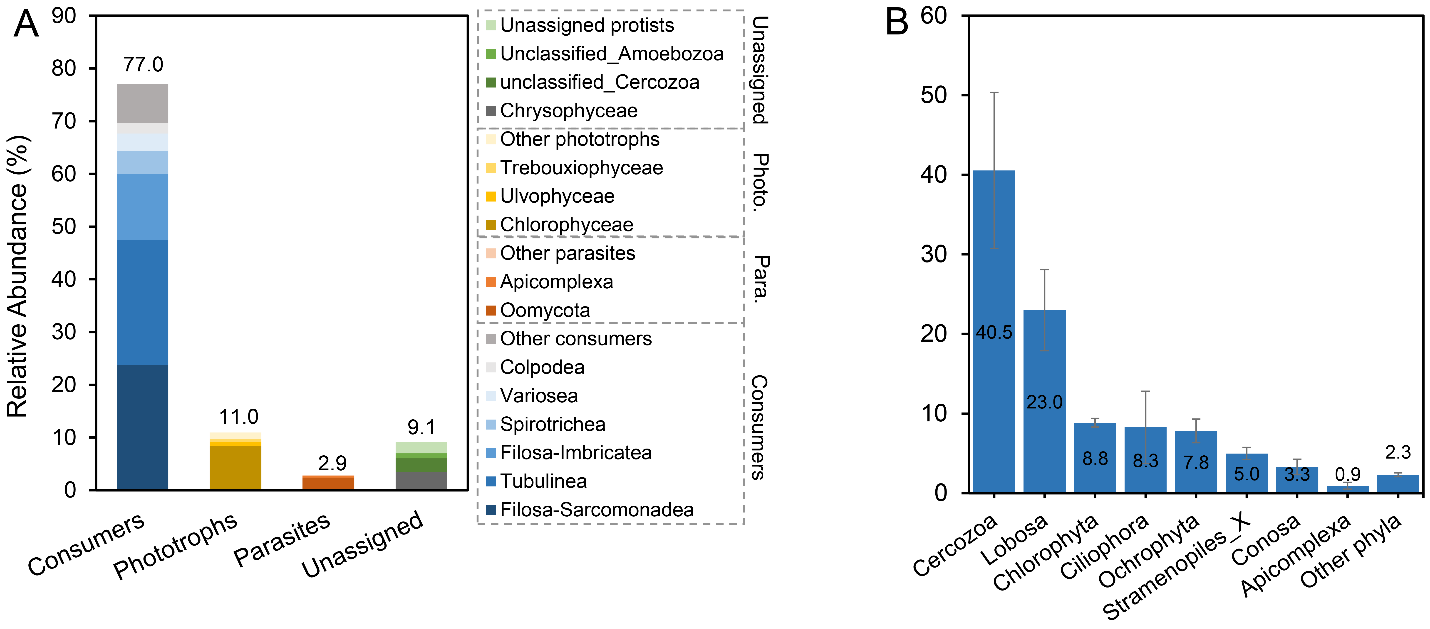


**Figure S3.** (A) The absolute abundance of protists (18S rRNA gene copies per gram soil) at different time points. (B) Alpha diversity of protists at different time points. (C) Relative abundance of bacteria at the class level in raw soil. (D) The absolute abundance of bacteria (16S rRNA gene copies per gram soil) at different time points. Letters denote the significant differences in the absolute abundance of protists or bacteria among treatments in each time point (one-way ANOVA, post hoc LSD test, *p* < 0.05).


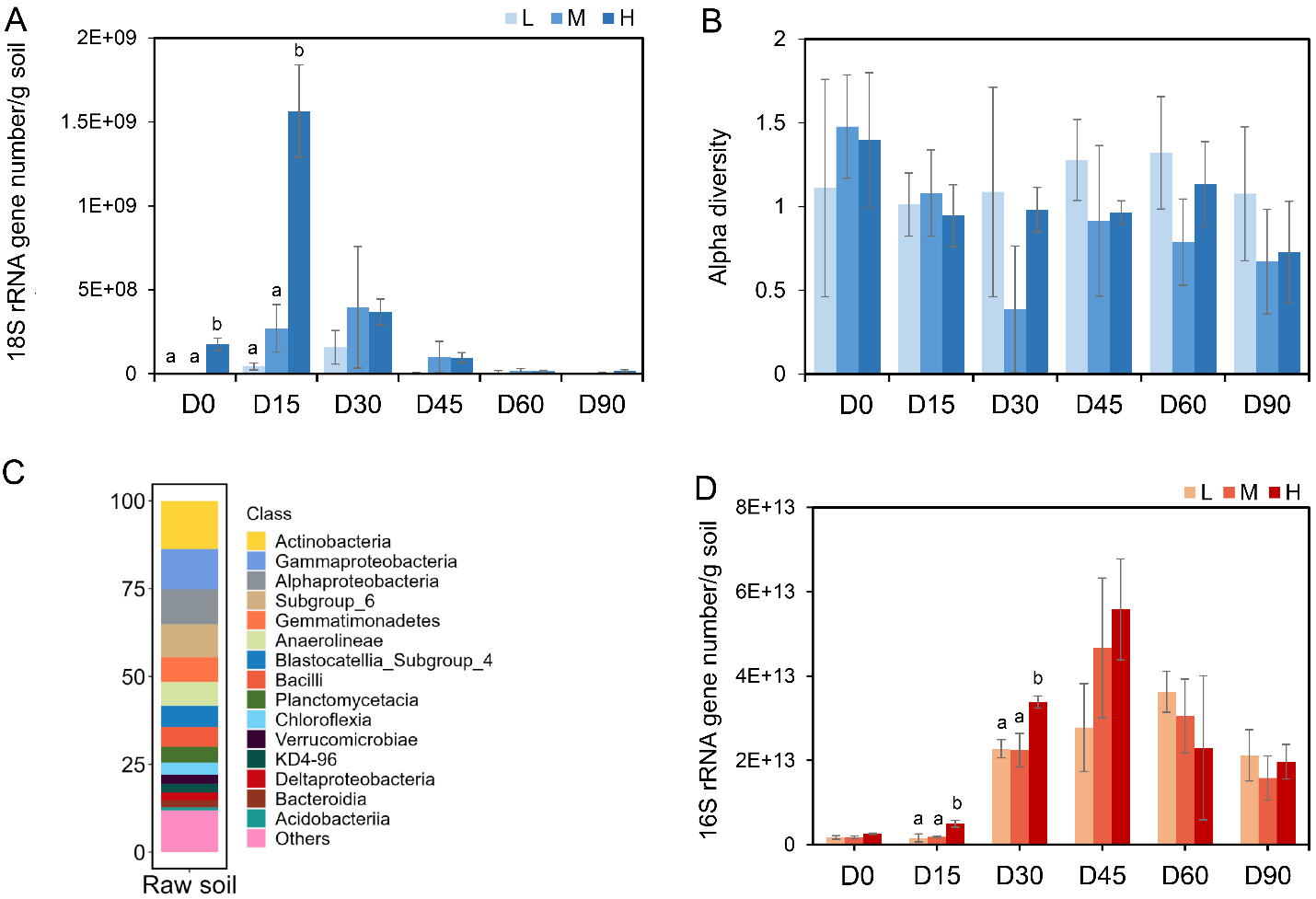


**Figure S4.** Relative abundance of the top 60 most dominant bacterial taxa at the class (left) and genus (right) level. (Abbreviations: L = low; M = medium; H = high protist concentrations).


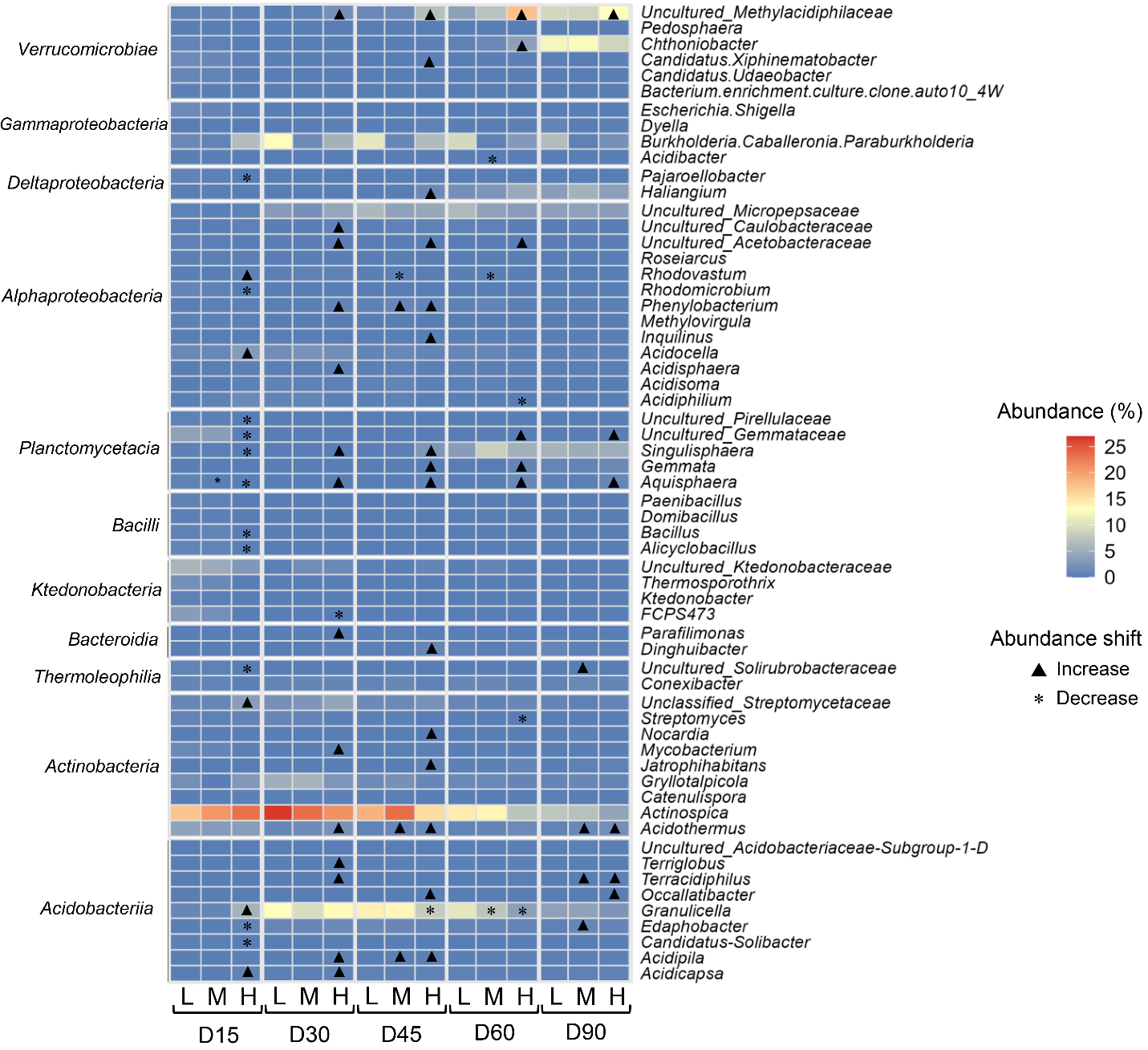


**Figure S5.** (A) Table showing the number of bacterial genera and bacterial – bacterial connections which strongly correlated to each other in the low, medium and high protist treatments. (B) The percentage of positive and negative connections among bacteria at different protist treatments over time. These connections were based on Spearman’s correlations among bacterial taxa in each treatment across all time points.


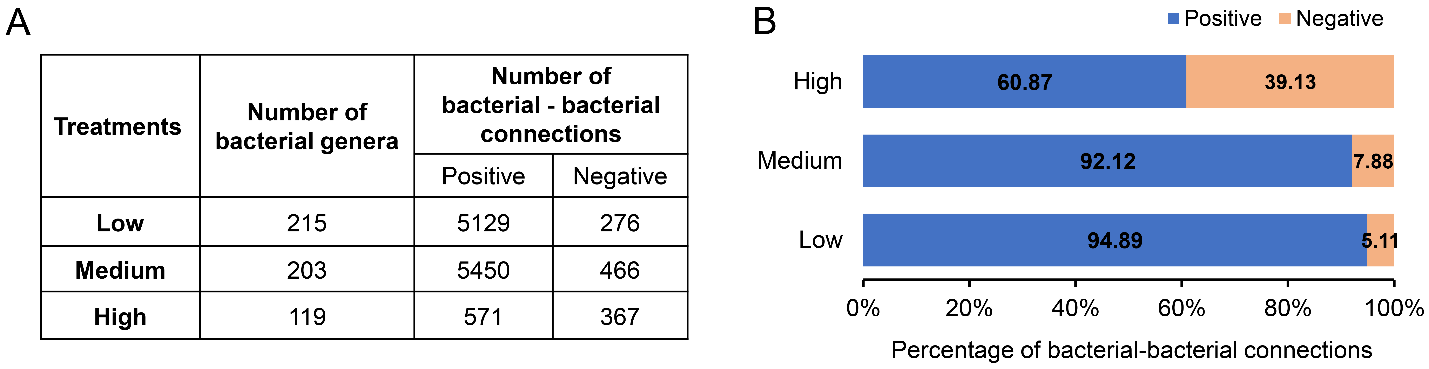


**Figure S6.** Relative abundance of the top 50 most abundant antibiotic resistance genes (ARGs) belonging to common antibiotic classes in different samples and at different time points. (Abbreviations: L = low; M = medium; H = high protist concentrations).


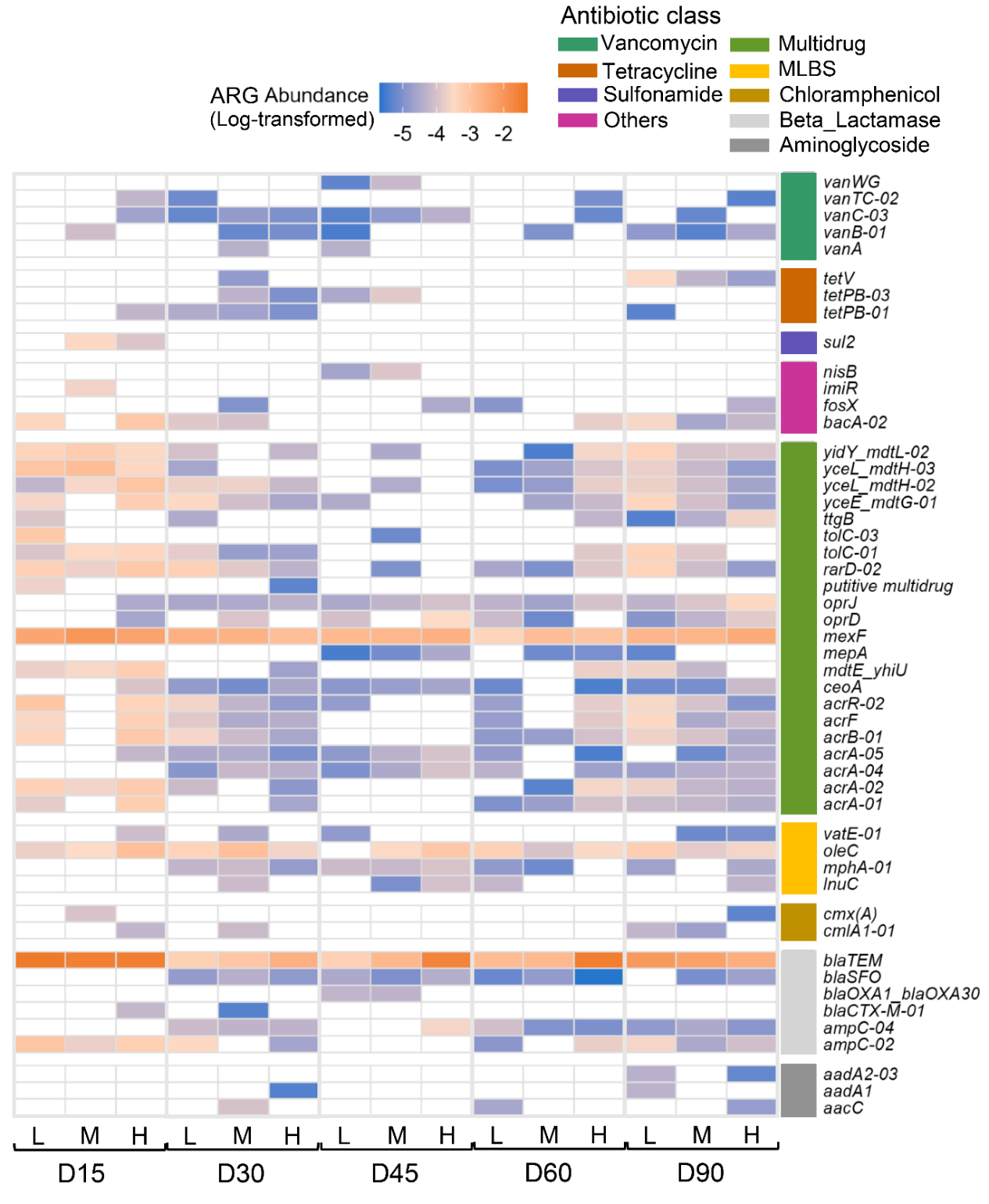


**Figure S7.** The ARG abundance (A) and richness (B) at different time points. Letters indicate the significant difference in the ARG number among three treatments (one-way ANOVA, post hoc LSD test, *p* < 0.05). (Abbreviations: L = low; M = medium; H = high protist concentrations).


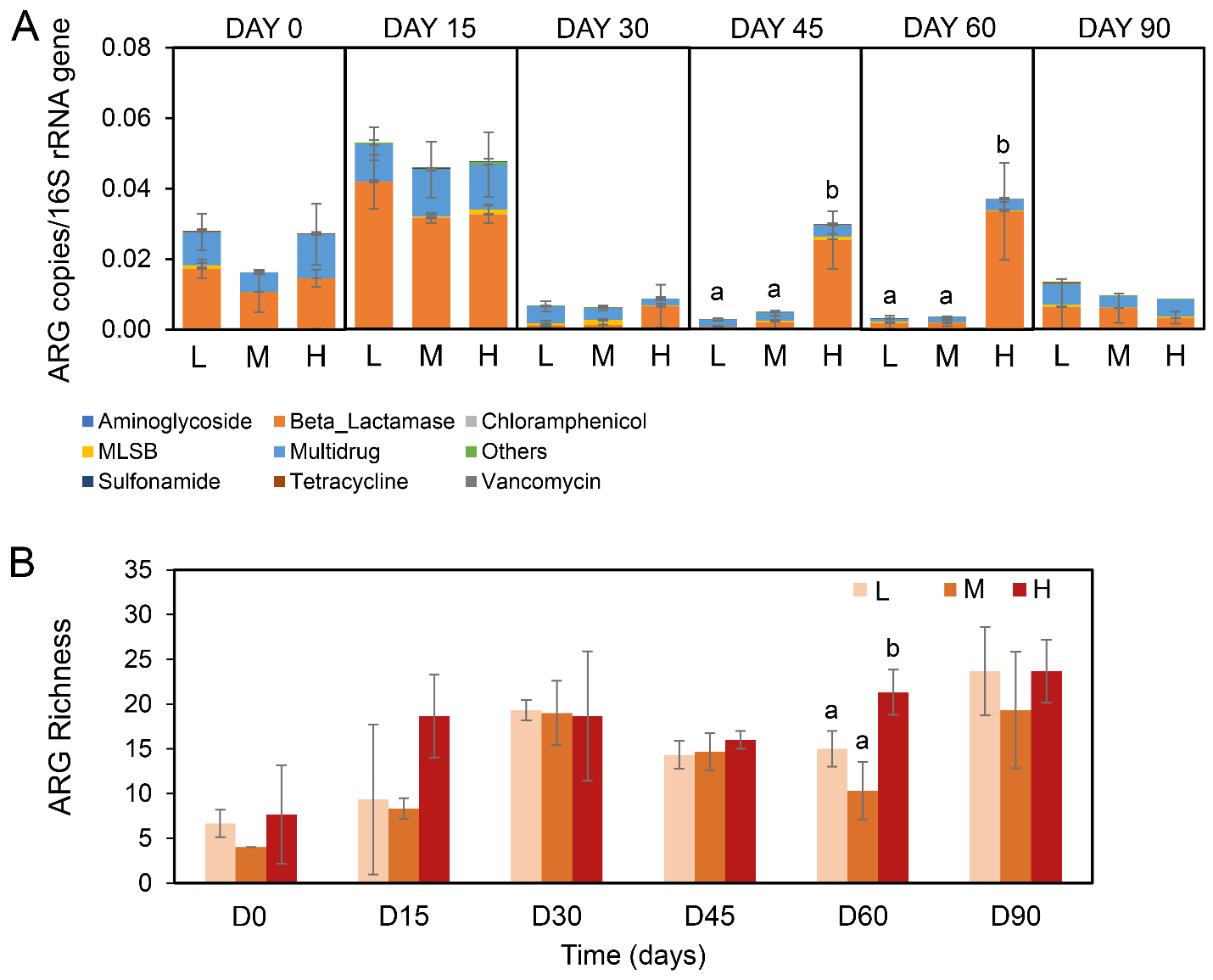

Supplement: Supplementary file 1 — Supplementary file [file 41396_2023_1524_MOESM1_ESM.docx]
