# Supplementary figures and images for: Protistan predation selects for antibiotic resistance in soil bacterial communities
Source: ISME J. 2023 Oct 4;17(12):2182–9. doi: 10.1038/s41396-023-01524-8 (PMC10689782; doi:10.1038/s41396-023-01524-8)

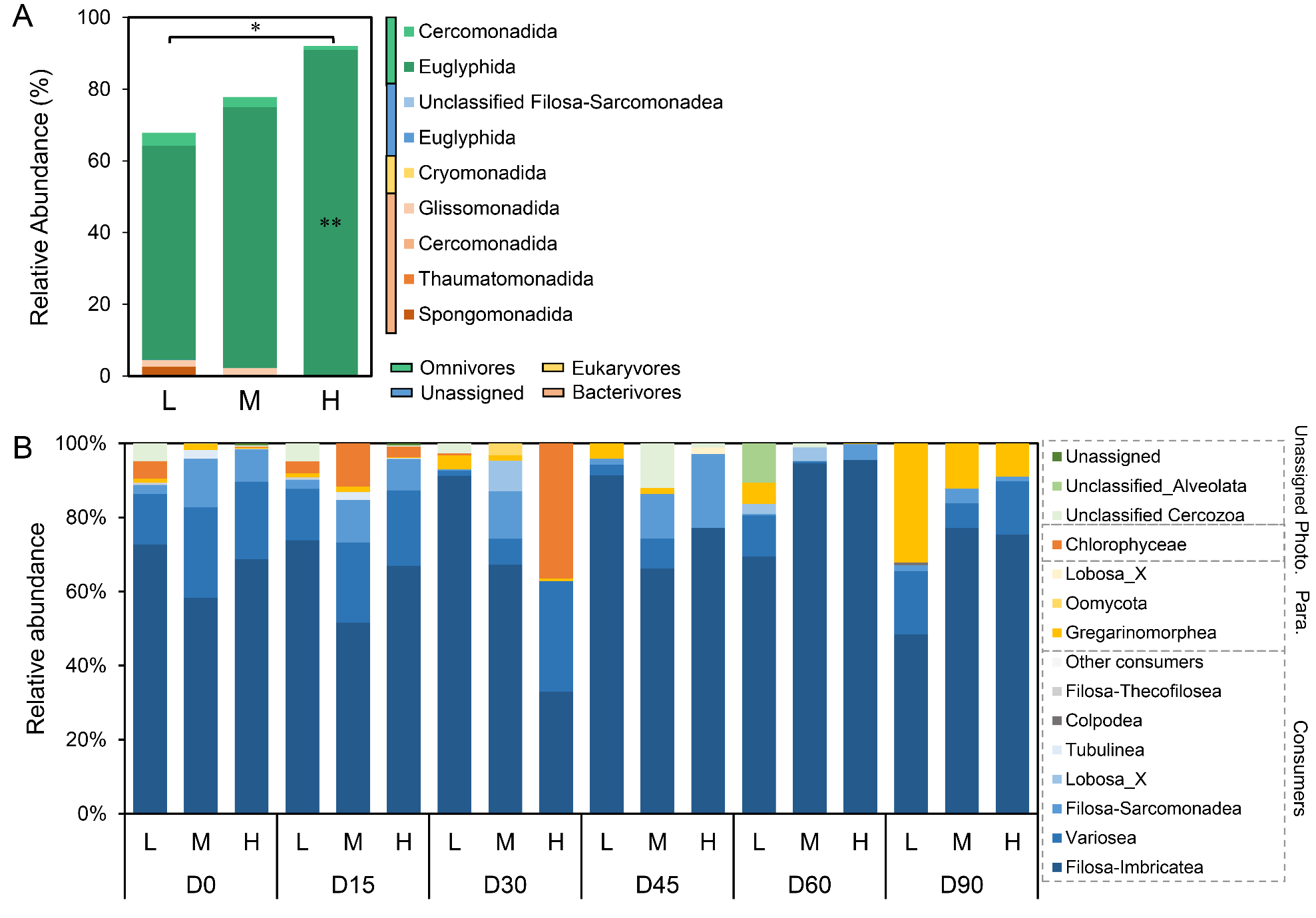

Supplement: Supplementary file 2 — Figure S1 [file 41396_2023_1524_MOESM2_ESM.docx]

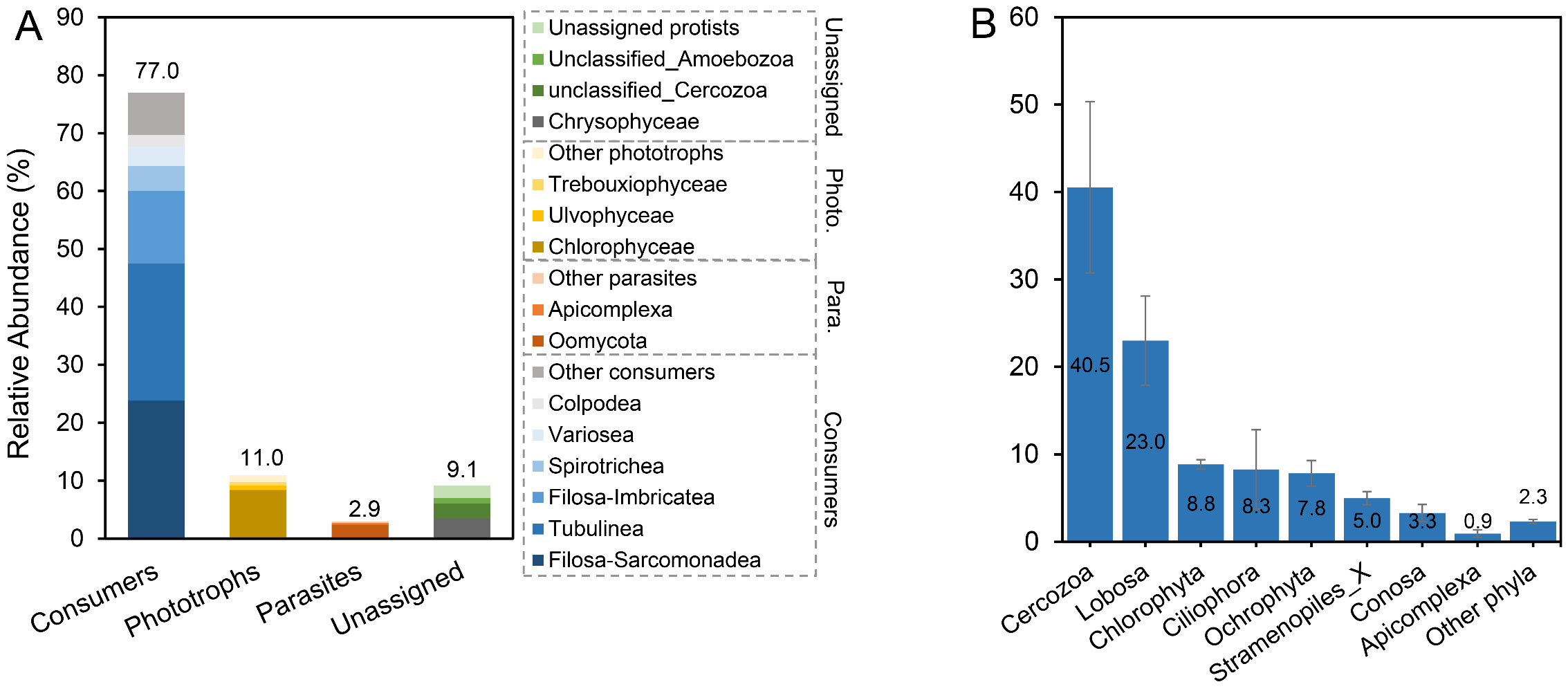

Supplement: Supplementary file 3 — Figure S2 [file 41396_2023_1524_MOESM3_ESM.docx]

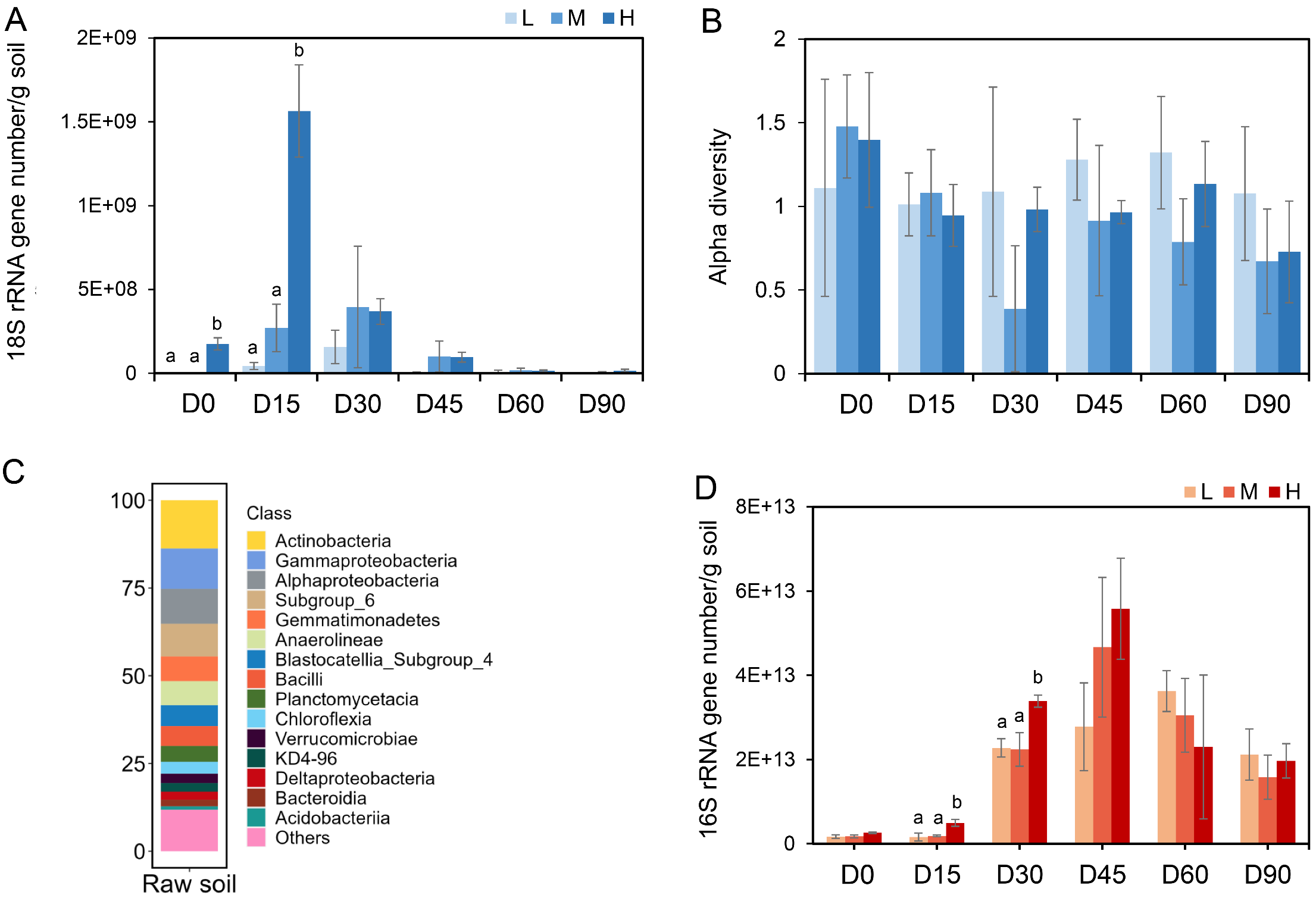

Supplement: Supplementary file 4 — Figure S3 [file 41396_2023_1524_MOESM4_ESM.docx]

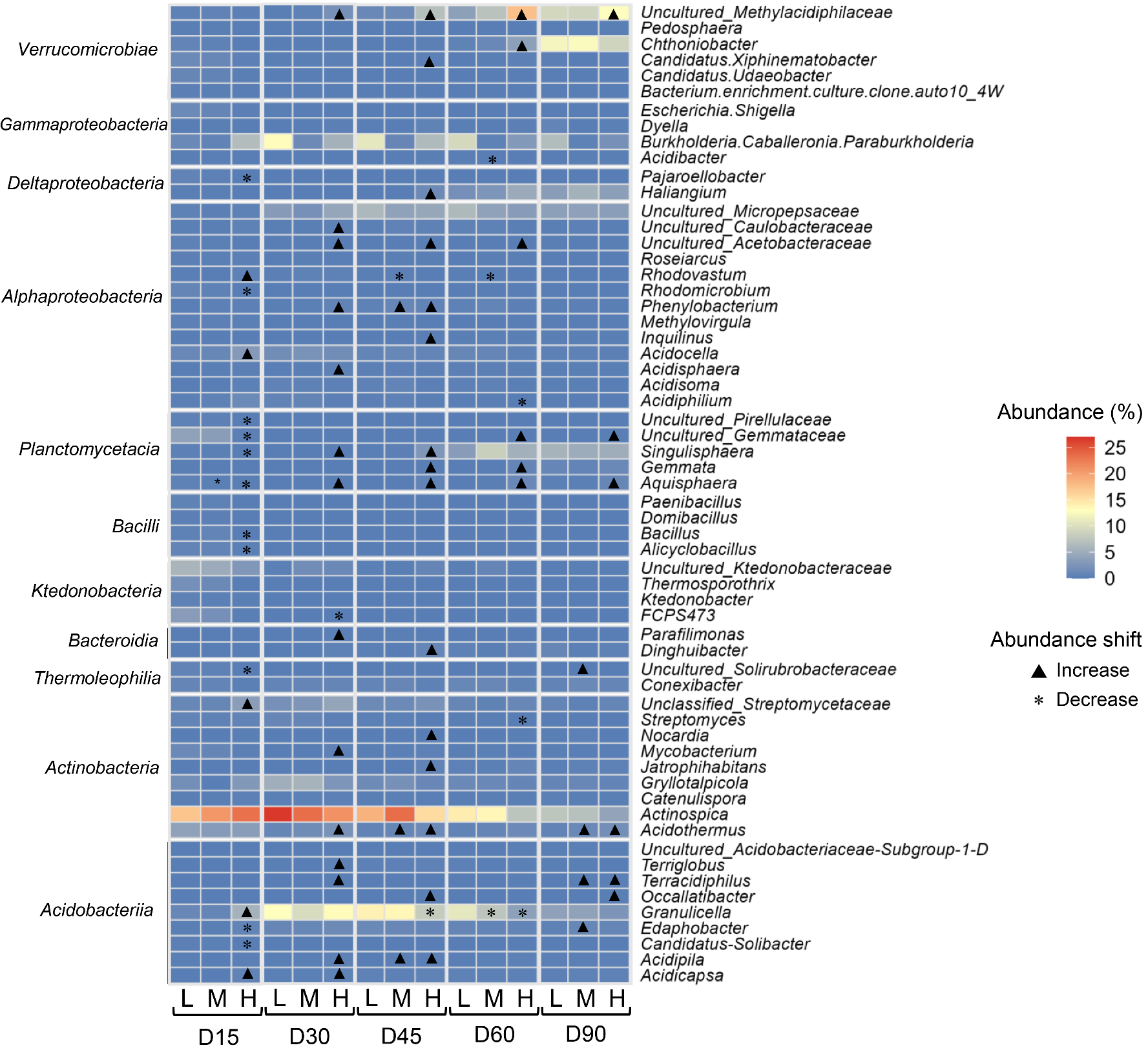

Supplement: Supplementary file 5 — Figure S4 [file 41396_2023_1524_MOESM5_ESM.docx]

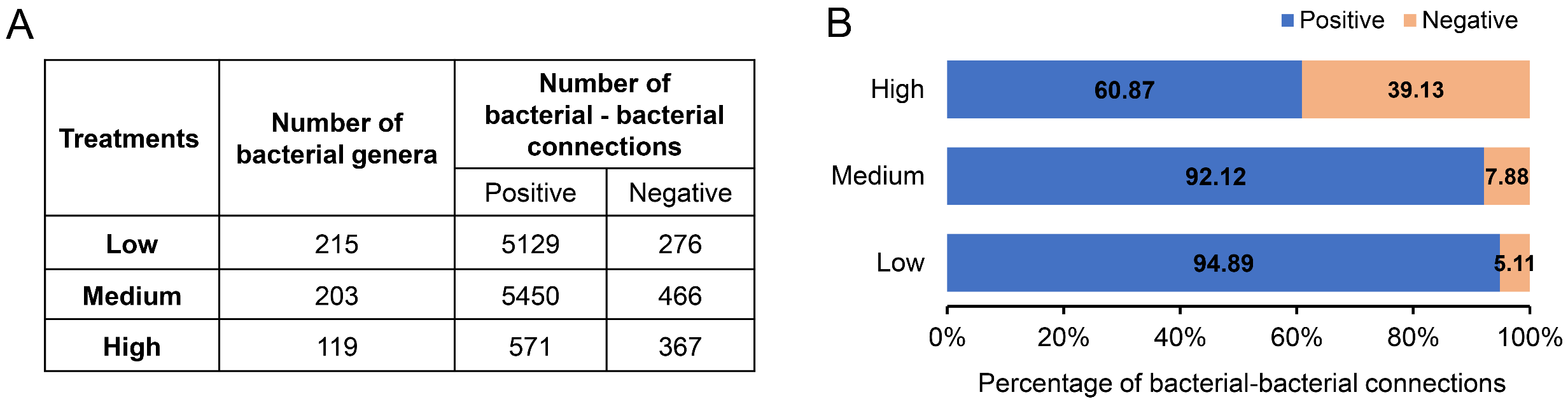

Supplement: Supplementary file 6 — Figure S5 [file 41396_2023_1524_MOESM6_ESM.docx]

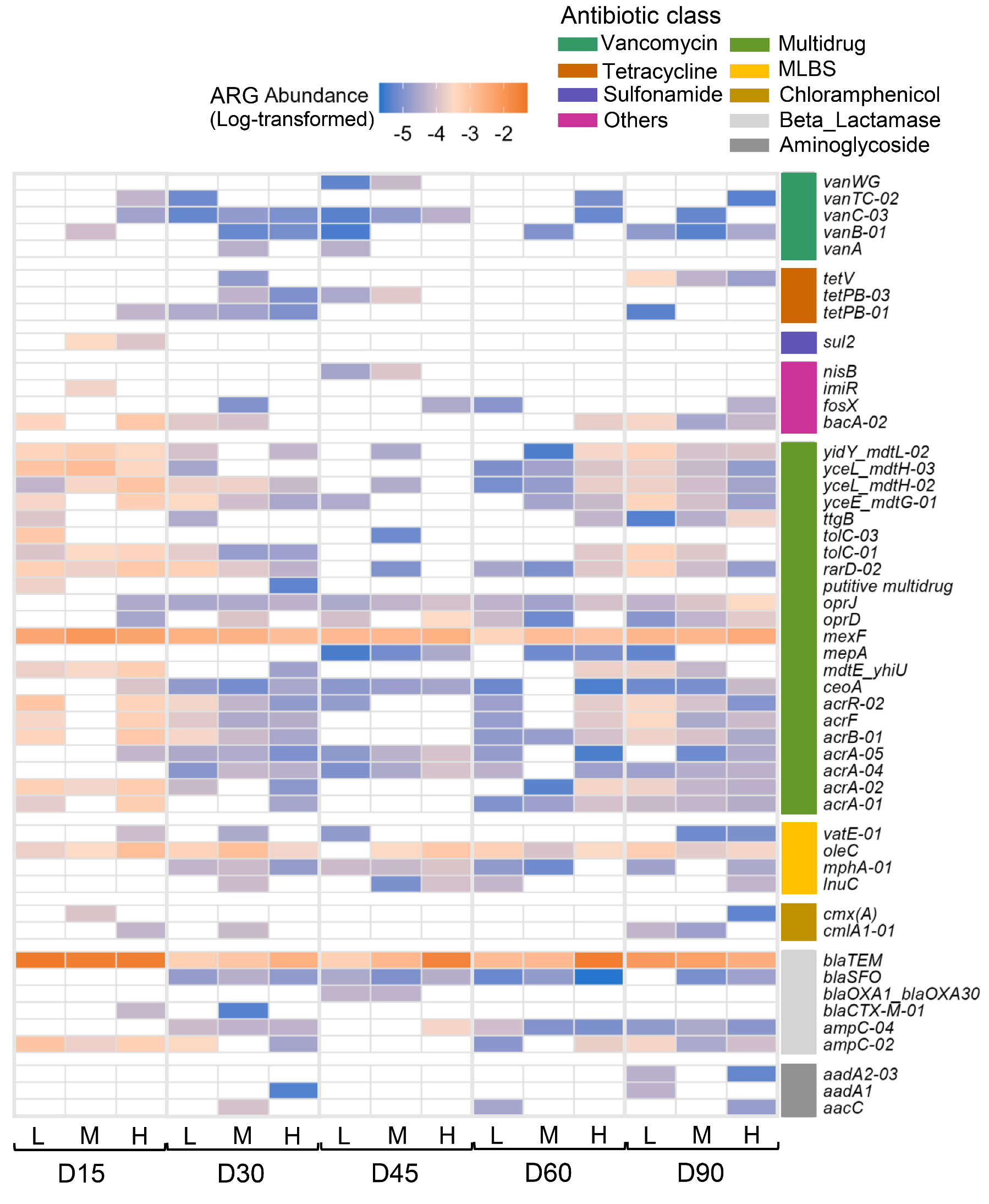

Supplement: Supplementary file 7 — Figure S6 [file 41396_2023_1524_MOESM7_ESM.docx]

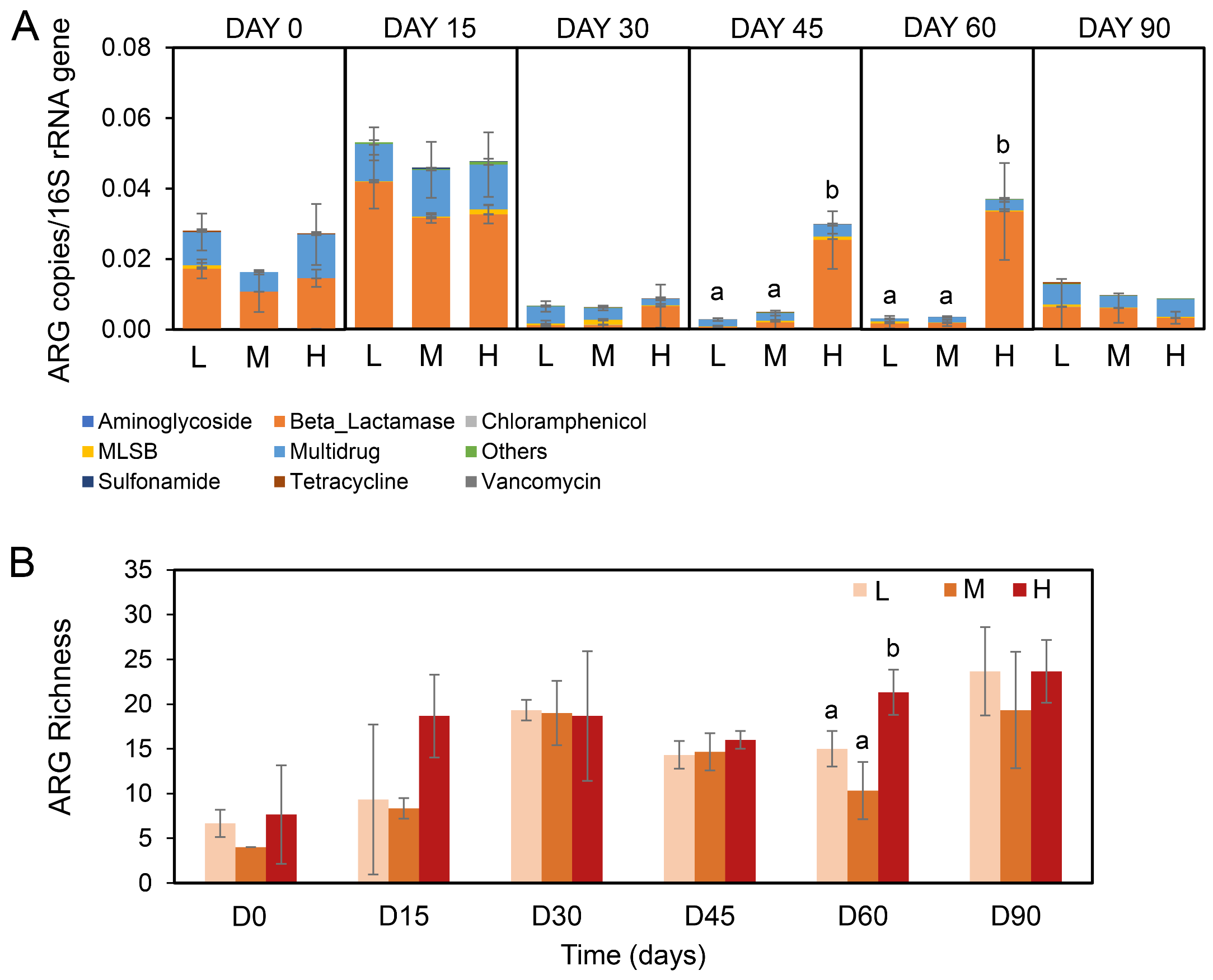

Supplement: Supplementary file 8 — Figure S7 [file 41396_2023_1524_MOESM8_ESM.docx]
